# OPEN, LAPAROSCOPIC, AND ROBOTIC-ASSISTED HEPATECTOMY IN RESECTION OF LIVER TUMORS: A NON-SYSTEMATIC REVIEW

**DOI:** 10.1590/0102-6720201700020017

**Published:** 2017

**Authors:** Túlio Felício da Cunha RODRIGUES, Bianca SILVEIRA, Flávia Pádua TAVARES, Gustavo Moreira MADEIRA, Iara Proença XAVIER, Jorge Henrique Costa RIBEIRO, Rayanna Mara de Oliveira Santos PEREIRA, Sávio Lana SIQUEIRA

**Affiliations:** 1 Federal University of Ouro Preto, Medicine;; 2 Department of Surgery, Gynecology, Obstetrics and Propedeutics, School of Medicine, Federal University of Ouro Preto, Ouro Preto, MG, Brazil

**Keywords:** Hepatectomy, Laparoscopy, General Surgery, Liver, Robotics.

## Abstract

**Introduction:**

Several factors have made hepatectomy an increasingly safe surgery and new drugs allowed surgical treatment for patients who initially were not candidates for resection. Lesions often require resection, which can be performed by open, laparoscopic, or robotic assisted hepatectomy.

**Aim:**

Compare the surgical techniques in open, laparoscopic, and robotic assisted hepatectomy for resection of liver tumors.

**Methods:**

Literature review based on scientific papers published on Lilacs/Pubmed/Scielo in the last 17 years regarding the indications of these techniques for liver tumor resections and on papers comparing such techniques.

**Results:**

The comparative study shows the benefits of laparoscopic surgery over open surgery, such as smaller incisions, less postoperative pain, shorter recovery time, smaller immune and metabolic response, and quicker restoration of oral ingestion as well as lower morbidity rates. However, the need for a specialized surgical team and the reduction in handling area still remain as disadvantages in the laparoscopic technique. It is yet not clear whether robotic assistance presents considerable benefits over the laparoscopic technique considering that high acquisition and maintenance costs are limiting factors.

**Conclusion:**

Despite all challenges, laparoscopic hepatectomy presents many benefits over open surgery. The robotic assisted technique is still in evolution as many centers in the world perform hepatic resections with the platforms but only after a thorough patient selection. Thus, laparoscopy stands as the best option, unless there is some contraindication to the procedure.

## RESUMO

**Introdução:**

Nas últimas décadas, inúmeros fatores transformaram as hepatectomias em operações mais seguras. A quimioterapia, juntamente com novas drogas para o tratamento de metástases propiciaram melhores respostas, o que possibilitou a indicação cirúrgica em pacientes que inicialmente não eram candidatos a ela. Lesões hepáticas muitas vezes requerem ressecção, que pode ser realizada tanto por laparotomia, por videolaparoscopia ou assistida por plataforma robótica.

**Objetivo:**

Comparar as técnicas cirúrgicas para ressecção de tumores hepáticos.

**Métodos:**

Trata-se de revisão com base em artigos científicos publicados nos últimos anos, sobre as indicações dessas técnicas e em artigos que comparam-nas. A pesquisa foi realizada nas bases de dados Lilacs/Pubmed/Scielo.

**Resultados:**

O estudo evidenciou vantagens da videolaparoscopia sobre a laparotomia, tais como menores incisões, redução na dor pós-operatória, menor tempo de recuperação dos doentes, dentre outras. No entanto, a necessidade de equipe especializada e a restrição na manipulação da área, consistem, ainda, em desvantagens consideráveis da técnica laparoscópica. Atualmente, ainda não está claro se o auxílio robótico demonstra vantagem substancial sobre a técnica laparoscópica, sendo o alto custo de aquisição e manutenção importante fator limitante.

**Conclusão:**

Apesar dos obstáculos e desafios, a hepatectomia laparoscópica demonstra vantagens sobre a laparotômica. A técnica assistida por robótica ainda está em evolução, sendo poucos os centros no mundo que a realizam nas ressecções hepáticas. Dessa forma, indica-se a laparoscopia, a menos que haja alguma contraindicação para sua realização.

## INTRODUCTION

Over the last years, several factors have made hepatectomy an increasingly safe surgery. A better knowledge of liver anatomy, the development of imaging techniques, a more complete preoperative assessment of the patient and his liver function with multidisciplinary work as well as the improvement in surgical and anesthetic techniques have contributed to this^28^.

According to Lopes-Junior et al. (2014), some strategies have been developed to increase the resection rate in patients with liver primary tumors and metastases. As chemotherapy evolved, new drugs were developed, mostly for treatment of metastases, providing better responses and allowing surgical treatment in patients who initially were not candidates for resection.

Hepatic lesions can be benign or malignant and both may require resection^3^. Benign lesions of the liver can be hemangiomas, adenomas, or focal nodular hyperplasia. Such lesions are generally asymptomatic, with resection being required only when they generate symptoms. However, adenomas, although benign, are at high risk of complications and, thus, must always be removed surgically. Malignant tumors of the liver may be divided into primary and secondary tumors. Among the primary ones, hepatocellular carcinoma represents 70-85% of neoplasms and requires surgical treatment^26^.

The National Cancer Institute (INCA) develops yearly an estimate of cancer incidence in Brazil. The Estimate/2014 doesn’t present data regarding liver and biliary tract cancer but highlights the importance of these lesions due to their high lethality and sensitivity to preventive actions, such as immunization coverage^4^. Epidemiological data regarding the city of São Paulo in 2014, released by the national public health system (SUS), showed that the incidence of primary liver cancer was 2.07/100,000 and the mean age of patients was 54.7 years, with a male/female ratio of 3:4:1.^13^

As for the workup, tests such as ultrasound, computed tomography (CT), magnetic resonance imaging (MRI), angiography, and biopsy can be performed to aid in the diagnosis of liver cancer.

Surgery is the best treatment for primary liver tumors without distant metastases and metastatic liver tumors in which the primary lesion was resected or is likely to be resected in a curative way. The indication for hepatic resection depends on the clinical conditions of the patient and the expected amount of remaining liver parenchyma, which should be around 10% of the patient’s body weight. In cirrhotic patients, only the ones with a Child A classification (early cirrhosis) are candidates for safe liver resection^5^.

Another possible treatment is liver transplant, but only a small proportion of patients are good candidates for it since the criteria is based on the size and number of tumors. Currently, transplants are indicated for small tumors - three or less nodes up to 3 cm each or single nodes up to 5 cm in which there was no invasion of blood vessels or for cases where the tumor cannot be completely removed or the liver is too far compromised^25^.

So, the aim of this article was to compare the surgical techniques in open, laparoscopic, and robotic assisted hepatectomy for resection of liver tumors.

## METHODS

This literature review is based on scientific papers published regarding the indications for open, laparoscopic, and robotic assisted hepatectomy in resection of liver tumors and on papers comparing these techniques. The research was conducted using the databases Lilacs/Pubmed/Scielo with selection of articles published in the last 17 years related to the topic.

## RESULTS

### Patient preparation

A correct preoperative prepare must take into account the nature of the liver disease, its severity, and the type of operation to be performed^23^.

The assessment of liver function is essential and the classification of Child-Turcotte-Pugh is the simplest form to do that. Even small liver resections are not possible in most patients with B or C stages of this classification^23^ (Table 1).

**TABLE 1:** Child-Turcotte-Pugh classification

	1 point	2 points	3 points
Encephalopathy	0	1-2	3-4
Ascites	none	slight	moderate
Bilirubin (mg/dL)	<2	2-3	>3
Albumin (g/dL)	>3.5	28-3.5	<2.8
PT prolonged (s)	1-4	5-6	>6
(INR)	<1.7	1.8-2.3	>2.3

Child’s A = 5-6 points; Child’s B = 7-9 points; Child’s C = 10-15 points

The presence of portal hypertension (esophageal varices, ascites, and splenomegaly with thrombocytopenia) should always be evaluated since it is a better predictor of bad prognosis than the Child-Turcotte-Pugh criteria in patients undergoing liver resections^23^.

The MELD (Model for End Stage Liver Disease) score, which uses INR, creatinine, and bilirubin levels, can also be used as a pre-surgical evaluation parameter. What favors the use of this score is the fact that it is continuous, being performed numerous times throughout patient care. A reduction in the score indicates that the patient’s condition has improved, being, thus, possible to recommend the surgical procedure once the MELD is below 10. When the MELD score is between 10 and 15, surgery should be performed with caution and only when strictly necessary. In people with a MELD score above 15, surgical procedures are not recommended^23^ (Table 2).

**TABLE 2:** MELD Score

3 month mortality according to MELD score					
MELD score	<=9	10-19	20-29	30-39	>=40
Hospitalized pt.	4%	27%	76%	83%	100%
Outpatient cirrhotic	2%	6%	50%		

MELD score = 10x[0,957x log e (creatinine) + log e (bilirubin) + 1.12 x log e (INR)] + 643

The preoperative preparation can also include fasting, indwelling urinary catheter, prophylactic antibiotics (cephalothin), and two peripheral venous access, varying among cases^21^.

### Hepatic resection by open surgery

The open operation is performed with the patient in a supine 15º Trendelenburg position with the right arm at a 90º angle. The surgical incision is made across the superior abdomen, following the curvature of the ribs. It can be bilateral subcostal for larger resections or only extended to the left for minor resections. When the mass to be addressed is very large, one can combine access between the abdomen and the chest^12^ (toracophrenolaparotomy, anterolateral thoracotomy associated with laparotomy). Calne (1968) popularized the bilateral subcostal incision with midline extension. The use of retractors is essential to keep the costal railings apart, which allows better visualization of the surgical field for long periods of time^12^.

The use of intraoperative ultrasound is essential to identify the location of the nodules during surgery as well as their relationship to the blood vessels of the liver. It is also possible to identify new nodules not visualized on CT or MRI^12^. 

It’s important to consider bleeding control methods, especially the Pringle maneuver (temporary compression of the portal triad: hepatic artery, common bile duct, and portal vein) and total hepatic vascular exclusion (interruption of the blood flow through the inferior vena cava supra and infrahepatically associated with clamping of the portal hilum). After vascular control, the compromised part of the liver is removed. At the end of surgery, a drain may be left near the area where the liver was cut to monitor bleeding and bile leakage^12^. 

The extent of the resection depends on the hepatic function of the patient. If there is no cirrhosis up to two thirds of the liver can be removed surgically. When lesions are large, patients must undergo, whenever possible, major liver resection, which is related to an increased progression-free survival^7^. In such case, the preoperative portal embolization of the lobe to be resected promotes hypertrophy of the remaining liver, making the resection safer and reducing the morbimortality rates^14, 2, 9^.

There is no consensus regarding the optimal surgical resection margins. Some studies show that segmental resections of any segment or sector where the tumor is located, including its portal pedicle, showed better results than enucleations but other papers do not demonstrate this superiority. Several studies show that resection margins larger than 1 cm are associated with higher survival rates^7^.

When there is macroscopic vascular invasion, resection is contraindicated, as this is known to be a poor prognostic factor associated with high recurrence and an overall survival rate of less than 10%^7^.

The surgery lasts 3-4 h and the patient must stay in the ICU (24 h) for monitoring of bleeding and liver function, returning to the ward when stable. After this, the patient may remain hospitalized for as much as 10 days. After surgical removal of part of the liver (in a normal liver up to 75% of the organ can be withdrawn), the remaining tissue starts to regenerate 48 h later and reaches a size similar to normal within 3-4 weeks. The function returns to normal in about 6-8 weeks^12^.

The most common clinical complications are pneumonia, deep vein thrombosis, pulmonary embolism, and liver failure. Regarding surgical complications, bleeding and bile leakage on the liver resected surface can occur. It’s important to note that liver resection has a high tumor recurrence rate of up to 50%. This may be related to metastasis of the resected tumor or to the appearance of new foci since the remaining hepatic parenchyma remains diseased. However, hepatic resection preserves the possibility of liver transplant, ablation techniques, or following resections in cases of recurrence^7^.

The most common contraindications for surgery are: patients with compromised cardiopulmonary function, severe malnutrition, impaired liver function, extrahepatic metastatic disease, and invasion of the portal vein bifurcation or of the hepatic veins trifurcation^12^.

Hepatic resection through open surgery was the treatment of choice for many years but was limited due to high rates of morbidity, mortality, and recurrence of the underlying liver disease^14^. Currently, liver resection can be performed in specialized centers, with less than 5% of mortality and up to 70% of five-year survival in selected patients (asymptomatic tumor and good liver function). However, tumor recurrence may occur in as much as 50% of cases within three years, even with proper patient selection^7^.

Some advantages of hepatic resection that can be cited are: a) immediate availability in specialized center; b) low risk in well-selected patients; c) precise histological evaluation; d) overall survival rates comparable to those with the intention of transplanting; e) possibility of rescue liver transplant in cases of relapse, as long as patients are monitored closely for early diagnosis of recurrences; and f) reduction of costs on the global economy of liver transplant^14^.

### Laparoscopic hepatic resection

The technical advances in laparoscopy have revolutionized surgical treatment for many diseases. In recent years, these advances have enabled video-assisted resection of solid organs such as the kidney and the spleen. However, some surgeries, such as liver resection, are still viewed with skepticism, since factors such as the transection of the parenchyma, the potential for intra-operative bleeding, and the risk of air embolism make the procedure a controversial theme^20^.

Laparoscopic hepatectomy is a difficult and laborious procedure, requiring the availability of proper equipment and professionals with technical training in advanced laparoscopic surgery and liver surgery^20^.

Most of the studies indicate this technique for resecting benign hepatic tumors or treating hepatic cysts. The best candidates would be young patients with superficial or peripheral benign tumors with indication of limited parenchyma resection. It is also recommended that the method be employed in resections of the anterolateral portions of the liver, segments II, III, IV b, V, VI, or of the left portion of the caudate lobe. Within these criteria, the results are encouraging, with minimal morbidity and no complications such as bleeding or air embolism^20^.

The technical standardization, with use of conventional surgery technology adapted for laparoscopy is, according to Buell et al. (2005), an important aspect to facilitate the procedure. Among the new technologies available, laparoscopic ultrasound transducers should be highlighted as well as the use of hand assisted equipment, which consists of a ring set on a small skin incision of about 7 cm connected to a plastic bag, through which you can reach into the abdominal cavity without leaking gas from the pneumoperitoneum. Fong et al. (2005) reinforces that, with the use of this technology, the procedure becomes easier and safer, the confidence in obtaining safe margins is increased, and the removal of the specimen is facilitated^27^.

Bleeding is the major complication and the biggest challenge intraoperatively. Most conversions to open surgery, about 70%, happen due to intraoperative bleeding. Proper patient selection, meticulous technique, and clamping methods of the hepatic pedicle can reduce this dreaded complication. Hence, vascular control is a major concern in hepatectomies, especially in resection of tumors close to large vessels or in major resections. The clamping of the hepatic pedicle, Pringle maneuver, can be done easily by placing a shoelace around the hepatic hilum, which creates a tourniquet to control the hepatic flow^8^.

Another concern is the possibility of spreading tumor cells during removal of the fragment. The specimen should always be introduced into a sturdy material bag and its removal should be done through the umbilical incision, when it has less than 3 cm, or through an appendectomy or suprapubic incision, when it is a larger piece. There is no doubt that for the treatment of benign tumors, especially liver adenomas that affect young women, laparoscopic hepatectomy has an important role. The whole debate should be reserved for cases of cancer in which laparoscopic surgery could increase the risk of neoplasic cells implantation, a fact that has not yet been demonstrated^8^.

### Robotic hepatic resection

Robotics was introduced in medicine nearly two decades ago as a way of overcoming the limitations of movement of the laparoscopic instruments and providing better visualization of the surgical field. The Da Vinci station, the most used equipment for this type of surgery, was introduced in 2000. It has a three-dimensional view camera that allows a better sense of depth and is capable of more movements than the human hand is naturally capable of^22^.

In robotic assisted surgery, the patient is placed in a supine Trendeleburg position with legs apart. Commonly, five entrance points are positioned along a semicircular arc. The procedure then follows three phases: portal dissection, liver mobilization, and parenchyma transection. The main surgeon sits in front of the robotic device as the assistant surgeon stands next to the patient’s right side^17^.

In the first phase, the liver is retracted cephalically to expose the hilar region. The portal pedicle is dissected and its components are exposed. How the procedure will continue depends on which segment will be removed. Thus, the respective branches of the portal vein and hepatic artery are divided. The liver is mobilized after transection of the falciform and triangular ligaments, but other structures can also be sectioned depending on the procedure^17^.

After these steps, the capsule is incised through cauterization and the hepatic trasection begins. Two robotic arms can make the transection easier by retracting the liver and opening the surgical site. The other two arms carry a device of ultrassonic dissection and a diathermic scissor. Then a titanium clip or an endostapler is positioned to clamp the main vascular branches and biliar ducts. After the parenchyma transection is complete, the surface is inspected for any bile leakage or exudation. The specimen is then obtained through a Pfannenstiel incision inside a bag called endobag^17^

Robotic technique can also occur with fluorescence guidance through application of indocyanine green that can be injected into the bloodstream and becomes fluorescent once it gets in contact with light of a specific wavelength near the infrared spectrum (around 820 nm) or a laser beam. The fluorescence can be detected inside specific rooms and chambers and then transmitted to a standard monitor that allows the identification of anatomical structures in which the dye is present^6^. This fluorescence images system associated to robotics offers additional information to the surgeon regarding anatomy, blood perfusion, lymphatic drainage, and functional liver reserve. The technique could become standard in the future, considering its different diagnostic capacities.

The robotic platform has proven to be an effective tool, particularly in the urological and gynecological field. The major drawback so far are the high costs and the difficulties in providing on board training for surgeons. Many centers in the world have performed liver resections using the Da Vinci platform but only in highly selected patients^22^.

### Comparison of techniques

Among laparoscopic surgeries, hepatectomies were one of the last to be carried out. The surgery was viewed with some skepticism due to concerns about bile leakage, incomplete resection, and, particularly, bleeding control, as blood loss is almost inevitable in the resection of liver tumors. Moreover, hilar dissection, liver mobilization, and parenchyma transection demand advanced technical skills and an accurate knowledge of anatomy, being, thus, potentially more dangerous than other laparoscopic procedures^10^.

Despite the many obstacles and challenges, laparoscopic hepatectomy shows numerous benefits over open hepatectomy. These are the same as in all laparoscopic surgical procedures, among which we can highlight the fact that the patient reports less pain postoperatively, has a lower incidence of ileus, less scarring, a shorter recovery time and hospital stay, and a lower rate of complications^18^.

A case-control study conducted by Lee et al. in 2007 helps support this view since it showed that laparoscopic hepatectomy caused less blood loss, lower morbidity rate, and less overall operative complications. In another study conducted in 2009, Zhang et al. observed 78 patients who underwent laparoscopic hepatectomies. The procedure was successful in all patients, without conversion to open procedures, and only four required blood transfusions.

Fonseca et al. (2003) and Machado et al. (2013) conducted studies with, respectively, 57 and 107 patients who underwent laparoscopic hepatectomies. Blood transfusion was required in 29.8% and 18.7% of patients, respectively. There was no operative mortality in both studies. The rate of postoperative complications was 57.9% in the first study and 14.9% in the second. It is clear, therefore, that there has been an advance in the art of laparoscopic hepatectomy, as the percentage of complications and the volume of blood loss have been decreasing.

Alhomaidhi et al. (2012) conducted a comparative study through a literature review that showed that, between 1990 and 2010, 751 open hepatectomies and 4207 laparoscopic hepatectomies were conducted. On average, it took 65 min less to perform the laparoscopic surgery. Blood loss volume was 260 ml in patients undergoing laparoscopic hepatectomy, while the loss at laparotomy was of 1290 ml on average. Postoperative morbidity was relatively low on both operations. The length of stay and mortality were also reduced.

Franken et al. (2014) conducted a comparative study of 104 patients, in which 52 underwent laparoscopic hepatectomy (group 1) and 52 underwent open hepatectomy (group 2). They noted that the estimated blood loss average was lower in patients undergoing laparoscopy (237 ml) than in those undergoing open surgery (387 ml). The average hospital stay was five days in group 1 and six days in group 2. Although blood loss decreased with laparoscopic hepatectomy, the need for blood transfusion was not significantly different and the severity of complications was not different between the groups.

Koffron et al. (2007) conducted a study in which 300 laparoscopic liver resections were compared to open procedures. They noted that the laparoscopic approach was superior to the open technique. The benefits were significant in the following aspects: duration of surgery (99 vs. 182 min), blood loss (102 vs. 325 ml), need for transfusion (two out of 300 cases vs. eight out of 100 cases), hospitalization (1.9 compared to 5.4 days), overall postoperative complications (9.3 vs. 22%), and local recurrence of malignancy (2 vs. 3%).

Wakabayashi et al. (2014) and Wang et al. (2015) also conducted comparative studies indicating that laparoscopic hepatectomy is better than the open technique. It was noticed that the exposure of the region is better, the hospital stay is shorter, the size of the incision and the blood loss was smaller, and the cost to the hospital is lower. Despite all these advantages, it was elucidated by Wakabayashi et al. that the restriction in the handling area is a considerable disadvantage. Wang et al. also noted that the possibility of tumor dissemination and the difficulties in maintaining adequate margins are potential disadvantages. 

According to Machado et al. (2012), in patients with a previously identified technical difficulty for the exclusive use of laparoscopy, hybrid techniques may be used with hand assistance or laparoscopic release followed by liver section through a small incision. The use of hand assisted techniques makes it easier to display the liver, to section the parenchyma, especially in cirrhotic livers, and also allows the surgeon the tactile sensation lost in laparoscopy. However, it is believed that a frequent use of this technique is not required, as it should be a step prior to complete conversion to laparotomy, or an option when difficulties for the realization of a total laparoscopic technique are expected.

The literature review shows an exponential growth in the number of and indications for laparoscopic hepatectomies. In a review of all published cases of laparoscopic hepatectomies, held in 2009, in which 2804 cases were identified, the mortality rate was only 0.3% and morbidity 10.5%^12^. Thus, from the perspective of Pais-Costa et al. (2011), the questions initially raised about the use of laparoscopy for treatment of malignancies no longer persist and currently the procedure is carried out with enough frequency to be considered very safe and effective.

Therefore, if there is indication for a hepatectomy, provided there is no contraindication for the method (intestinal obstruction, generalized peritonitis, severe cardiopulmonary disease, or severe hypovolemic shock among others reasons), laparoscopy should be the technique of choice^20^.

When comparing laparoscopic and robotic techniques, in accordance with Montalti et al. (2015), it’s noticeable that, despite laparoscopy’s worldwide spread since its introduction, robotics have not had the same evolution, possibly due to the significant initial costs and the different levels of required learning. Studies show that there is no significant difference in morbidity rate between the two techniques, although it was observed a tendency for fewer complications in the robotic group.

In a systematic review and meta-analysis, Montalti et al. (2015) states that the robotic platform is a tool with which many of the limitations of conventional liver laparoscopic surgery could be overcome, like image amplification, two-dimensional tremor, fulcrum effect, limited freedom of movement, and ergonomics. Furthermore, the increased dexterity activated by the endowristed movements, the filter software for the surgeon movements, and the high definition 3-D vision provided by a stereoscopic camera allows a constant and careful dissection of structures. All of this leads to minimal biliary leakage and overall reduction in postoperative complications^22^.

Despite all these advantages, robotic assisted hepatectomy has evolved slowly over the years and it is currently unable to provide useful tools to fully exploit the potential of the movements and the vision offered by the robot, especially when the resection space is limited.

It is important to note that to perform the robotic technique, an additional surgeon responsible for the procedure is required and the costs with the robot, the instrumentation, and the annual maintenance are high. In addition, there are few centers in the world that perform robotic liver resections and they always go through a thorough patient selection, which limits the number of procedures performed, and keeps it as non-standard, even if these centers can surpass the learning curve.

The results of the meta-analysis made by Montalti et al. (2015) show a significant rise in bleedings during robotic assisted hepatectomies, which can be explained by the different techniques used to execute the liver transection. The most used technique for laporoscopic hepatic transection requires the use of a harmonic scalpel for superficial liver trasection and Cavitron Ultasonic Surgical Aspirator for deeper cuts, to provide a more meticulous and precise parenchymal structure dissection. In turn, in robotic resection the technique is based on crushing fixation, requiring, in most cases, the use of an intermittent occlusion of blood flow (Pringle’s Maneuver).

Another important factor regards the operation time, which was significantly higher in robotic hepatectomy. This may be due to the technique itself buy may also be a result of robotics being new and requiring, therefore, greater experience and refinement.

As it is known, one of the principles of oncologic resection of malignant tumors is the maintenance of free margin to avoid incomplete resection of the tumor and possible iatrogenic spread. That is why the previously mentioned meta-analysis aimed to observe the margins width in each technique. As a result, no significant difference was observed, although there was a tendency for smaller margins in laparoscopic hepatectomies, which suggests a greater difficulty in identifying the lesion through robotic intraoperative ultrasound. This can be explained by the fact that the surgeon who performs the ultrasound is not the one responsible for the robotic hepatectomy. However, further studies are necessary for a better data analysis. The study of Montalti et al. (2015) considers that laparoscopic hepatectomies have a reduced blood loss and a shorter operative time compared to robotic hepatectomies.

Thus, it is not clear whether robotic assistance demonstrates substantial advantage over laparoscopic techniques since both approaches are considered minimally invasive, with no differences in safety or efficacy^22^.

## CONCLUSION

The approach of liver tumors is considered complex as it involves factors related to the clinical condition of the patient, the function of the liver, and the stage and characteristics of malignant diseases. Some tumors develop in livers considered normal, while others arise in organs compromised by obstruction of the biliary tract or liver diseases such as steatosis, fibrosis, or cirrhosis. Each of these factors can influence the result of surgeries and, therefore, careful attention to all of these aspects must be paid in order to achieve good results. The laparoscopic approach is more beneficial when compared to the open technique, despite the barriers and mistrust that still remains. Laparoscopy has shorter procedure duration, shorter hospital stay, and lower incidence of complications. Furthermore, the local malignant recurrence rate is also reduced in the closed surgical procedure.When laparoscopic and robotic techniques are compared, it is clear that the robotic assistance can overcome many limitations that laparoscopic surgery presents. However, due to the high cost and different levels of learning required, robotic hepatectomy still hasn’t spread worldwide. It is not yet clear whether robotic assistance is an advantage over laparoscopy, since hospital stay, morbidity, and estimated blood loss are similar. 
